# Isolated Squamous Morular Metaplasia of the Endometrium in the Absence of Proliferative Pathology: A Case Report and Review of the Literature

**DOI:** 10.7759/cureus.107452

**Published:** 2026-04-21

**Authors:** Jessica Evans, Wanda I Torres

**Affiliations:** 1 Dr. Kiran C. Patel College of Osteopathic Medicine, Nova Southeastern University, Clearwater, USA; 2 Obstetrics and Gynecology, Suncoast Women's Care, Trinity, USA

**Keywords:** endometrial hyperplasia, endometrial proliferative spectrum, endometrioid adenocarcinoma, isolated squamous morular metaplasia, wnt/β-catenin pathway

## Abstract

Isolated squamous morular metaplasia is an uncommon histopathologic finding in the endometrium that most frequently occurs in association with endometrioid proliferative lesions, including endometrial hyperplasia, endometrial intraepithelial neoplasia, and endometrioid adenocarcinoma. Squamous morular metaplasia in the absence of proliferative pathology is rare, and its clinical significance and optimal management remain poorly defined. A 43-year-old nulliparous woman presented with abnormal uterine bleeding. Her medical history was significant for morbid obesity and early menarche. Endometrial biopsy demonstrated focal squamous morule formation without evidence of endometrial hyperplasia or malignancy. A levonorgestrel-releasing intrauterine device was placed for the management of abnormal uterine bleeding and reduction of endometrial hyperplasia risk. Repeat endometrial biopsy revealed inactive-pattern endometrium, confirming stability with conservative progestin therapy. Studies have reported an association with isolated squamous morular metaplasia and subsequent endometrioid carcinoma and Wnt/β-catenin pathway dysregulation, supporting the need for clinical monitoring. In patients with significant risk factors for unopposed estrogen exposure, isolated morules may represent a marker of an endometrium predisposed to future proliferative changes. Isolated squamous morular metaplasia is an uncommon diagnosis that may warrant clinical surveillance due to its association with endometrioid carcinoma. Progestin-based therapy may provide a reasonable management strategy while allowing continued monitoring. Additional research is needed to clarify the prognostic value of morular metaplasia and the role of immunohistochemical evaluation in guiding follow-up strategies.

## Introduction

Squamous morular metaplasia (SMM) is a benign histopathologic finding in endometrial tissue characterized by the replacement of normal glandular epithelium by rounded aggregates of squamoid cells known as morules [1,2]. These structures typically appear as well-circumscribed nests of epithelial cells lacking the normal glandular architecture of the endometrium [1].

Morular metaplasia is most commonly encountered in association with endometrioid proliferative lesions, including endometrial hyperplasia (EH), endometrial intraepithelial neoplasia (EIN), and endometrioid adenocarcinoma (EAC) [2,3]. However, the presence of isolated SMM in the absence of concurrent proliferative pathology is uncommon. In a recent study, only 0.17% of endometrial samples were diagnosed with isolated squamous morular metaplasia [4]. Notably, endometrioid carcinoma was identified in 5.4% of patients with subsequent sampling or clinical follow-up [4]. Clinically, this distinction is important, as morular metaplasia is typically associated with premalignant or malignant endometrial conditions but may occasionally be identified in isolation.

Endometrial hyperplasia, endometrial intraepithelial neoplasia, and endometrioid adenocarcinoma represent a spectrum of estrogen-driven proliferative lesions with distinct histologic and clinical implications [3,5]. EH is defined as a disordered proliferation of endometrial glands and commonly occurs in the setting of unopposed estrogen exposure. Progressive architectural complexity, glandular crowding, and cytologic atypia may lead to the development of EIN, a premalignant precursor to one of the most common gynecologic malignancies, endometrioid adenocarcinoma [5]. EAC represents the malignant end of the proliferative spectrum and is characterized by neoplastic glandular growth that retains morphologic resemblance to endometrial glands [3].

Although most commonly associated with proliferative lesions, squamous morules may also arise in isolation, with proposed mechanisms including dysregulation of the Wnt/β-catenin pathway, which may drive altered endometrial epithelial differentiation in the absence of neoplastic changes. This signaling cascade plays a critical role in endometrial cell proliferation and differentiation as well as regulation of the menstrual cycle [6,7]. Under normal conditions, β-catenin is localized to the cell membrane and is targeted for degradation via the ubiquitin-proteasome system. However, mutations in the CTNNB1 gene, which encodes β-catenin, can result in nuclear accumulation of β-catenin and activation of downstream pathways that promote cellular proliferation. Alterations in this cascade have been described in endometrial hyperplasia as well as endometrial carcinoma [6]. In addition, nuclear β-catenin expression has been frequently described alongside morule formation in endometrial lesions [2,7]. 

Given the rarity of isolated SMM and its reported association with endometrioid carcinoma, the clinical significance of this finding remains uncertain [4]. Here, we report a case of isolated squamous morular metaplasia identified on endometrial biopsy in the absence of concurrent proliferative pathology, and discuss its potential clinical implications and management considerations.

## Case presentation

A 43-year-old nulliparous woman presented with abnormal uterine bleeding characterized by heavy menstrual bleeding lasting eight days associated with large clots and dysmenorrhea. Baseline menstrual characteristics prior to symptom onset were not clearly documented. Menarche began at age 11. Her medical history was significant for morbid obesity (body mass index (BMI): ~60 kg/m^2^), anxiety and depression treated with sertraline, and a long-standing smoking history averaging one pack per day.

Laboratory evaluation was unremarkable, including thyroid studies, prolactin, gonadotropins, androgen studies, insulin levels, fasting glucose, and a negative pregnancy test. Physical examination was unremarkable.

Transvaginal ultrasonography demonstrated a 2-cm subserosal uterine leiomyoma. Due to persistent abnormal uterine bleeding and concern for possible endometrial pathology, an endometrial biopsy (EMB) was performed.

**Figure 1:**
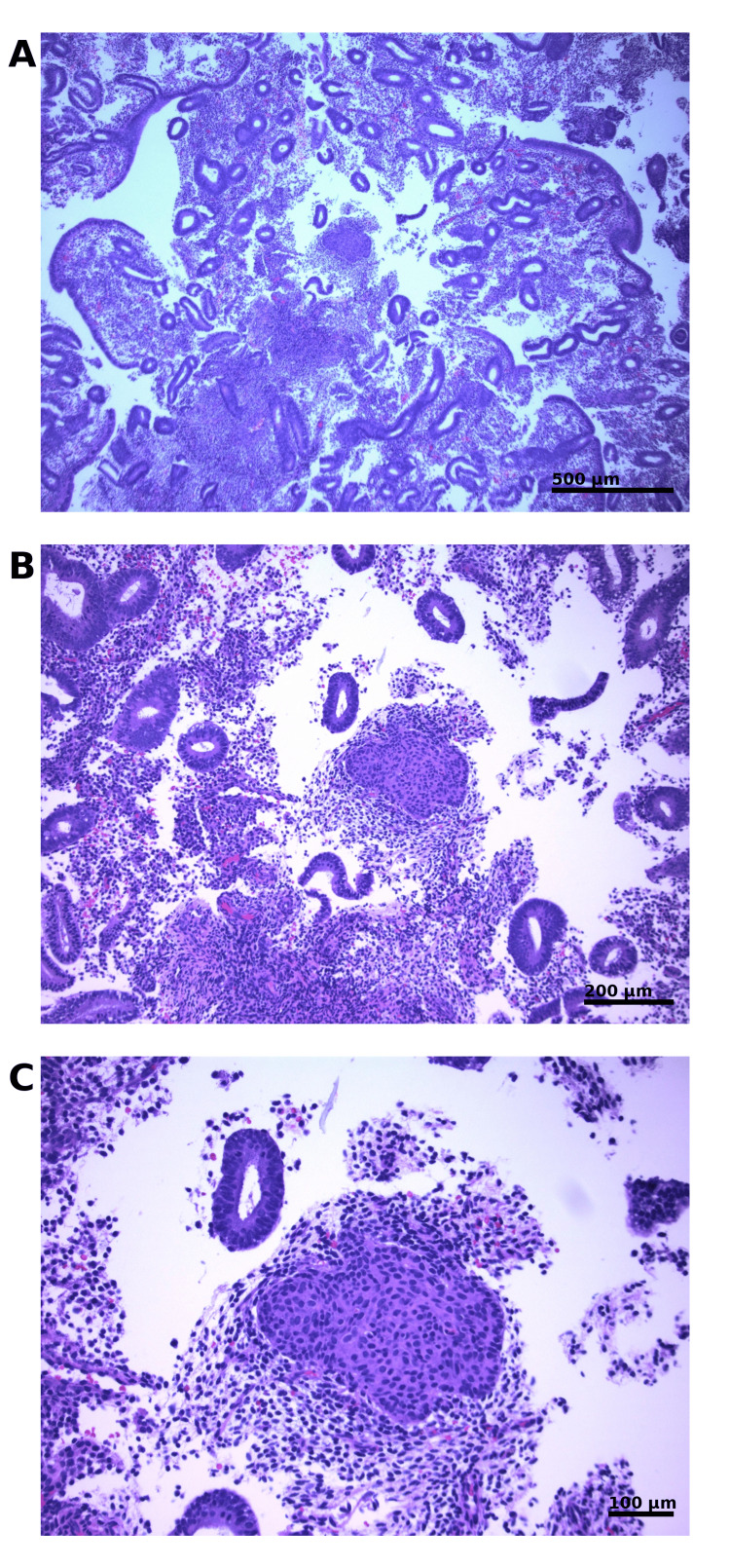
Histopathologic findings demonstrating squamous morular metaplasia. (A) Low-power view demonstrating a discrete morular structure within endometrial tissue (hematoxylin and eosin (H&E); 4×). (B) Intermediate magnification showing rounded aggregates of squamoid cells forming a morule (H&E; 10×). (C) High-power view highlighting bland cytologic features with eosinophilic cytoplasm and absence of cytologic atypia or keratinization (H&E; 20×).

Histopathologic examination using pipelle sampling demonstrated scant fragments of benign endocervical tissue and superficial squamous epithelium. Small fragments of interval-pattern endometrium were present and contained focal rounded aggregates of squamoid cells consistent with squamous morules (Figure 1A-C). The morules were composed of bland epithelial cells with eosinophilic cytoplasm and indistinct cell borders, without keratinization, cytologic atypia, glandular crowding, or architectural complexity. No evidence of endometrial hyperplasia, EIN, or malignancy was identified. Immunohistochemical (IHC) studies were not performed.

**Figure 2:**
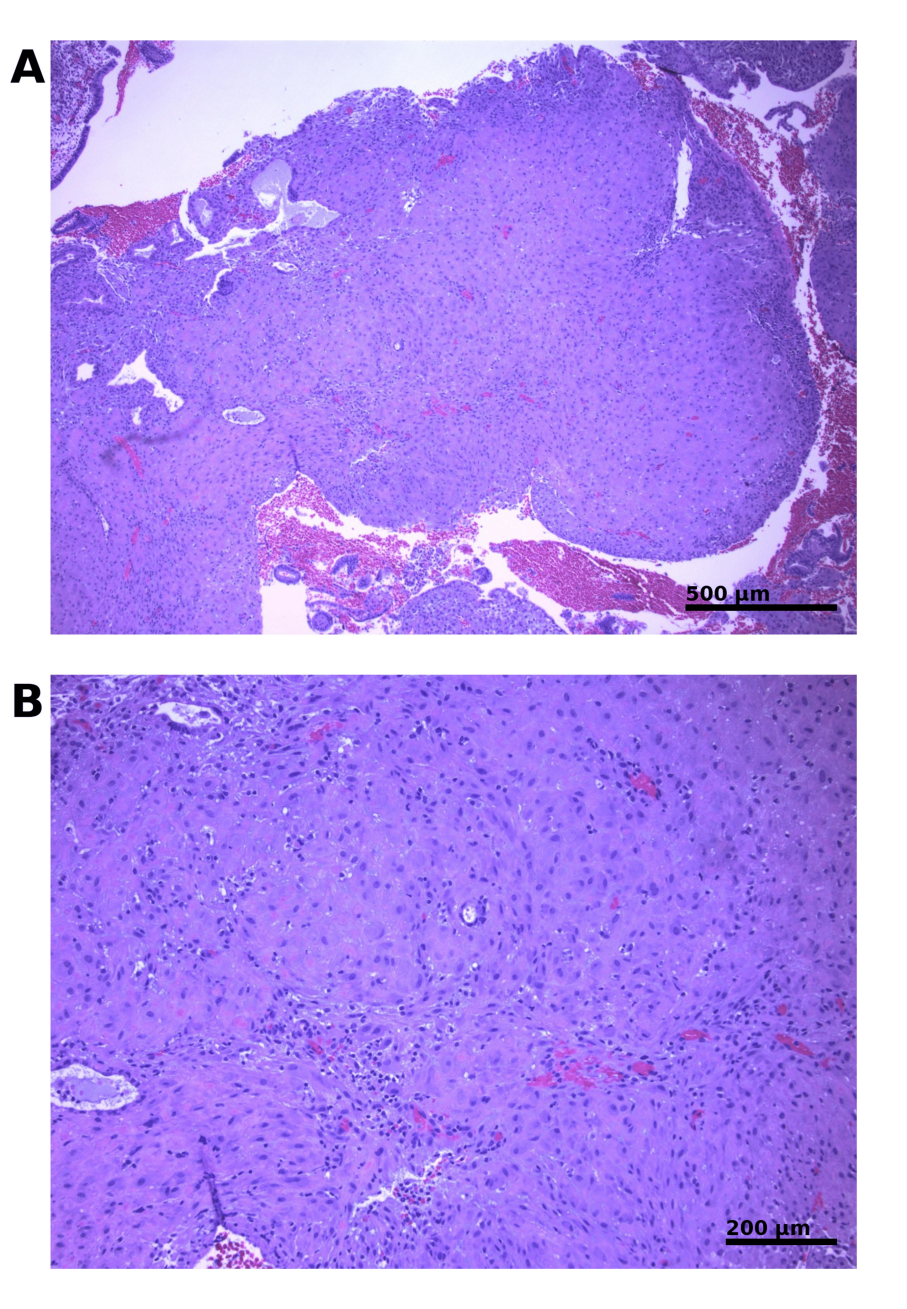
Endometrial biopsy following levonorgestrel-releasing intrauterine device placement. (A) Low-power view demonstrating inactive-pattern endometrium with stromal decidualization (hematoxylin and eosin (H&E); 4×). (B) Higher magnification showing glandular atrophy and absence of proliferative or atypical features (H&E; 10×).

A levonorgestrel-releasing intrauterine device (Mirena; Bayer, Whippany, NJ) was subsequently inserted for the management of abnormal uterine bleeding and reduction of endometrial proliferative risk. Follow-up endometrial biopsy revealed inactive-pattern endometrium without evidence of proliferative pathology (Figure 2A,B), confirming stability of the endometrium following conservative progestin therapy.

## Discussion

As previously stated, isolated squamous morular metaplasia is a rare entity, reported in only 0.17% of endometrial samples [4]. Previously reported cases of isolated morular metaplasia in endometrial biopsies are summarized in Table 1. Although morules are more commonly associated with endometrioid proliferative lesions, their presence in isolation presents a diagnostic and clinical challenge due to overlapping histologic features with premalignant and malignant conditions, limitations of endometrial sampling, and the absence of standardized management guidelines.

**Table 1:** Summary of studies evaluating morular differentiation and isolated squamous morular metaplasia in the endometrium. LNG-IUD: Levonorgestrel-releasing intrauterine device

Study	Year	Study Type	Cases	Key Findings
Chinen et al. [1]	2004	Pathology study	Multiple lesions	Morules identified in both benign and malignant endometrial lesions
Machin et al. [7]	2002	Molecular study	Endometrial carcinoma samples	CTNNB1 mutations associated with β-catenin nuclear accumulation
Niu et al. [2]	2022	Pathology cohort	Precancers/carcinoma	Morules correlate with CTNNB1 mutations
Zanfagnin et al. [4]	2026	Retrospective cohort	Isolated morular metaplasia	~6.5% developed carcinoma on follow-up
Present Case	2026	Case report	1	Stable endometrium after LNG-IUD therapy

The differential diagnosis of morular structures in endometrial biopsy specimens includes squamous differentiation within EIN or EAC, benign squamous metaplasia, and contamination by cervical squamous epithelium [1,3]. Now, reactive epithelial changes such as squamous metaplasia may occur in response to an infection or irritation; however, squamous morules are more specifically associated with dysregulation of the Wnt/β-catenin pathway and are most commonly seen in endometrioid neoplasia. In the present case, morule formation was identified in the absence of glandular crowding, cytologic atypia, or an architectural complexity, making EIN or atypical hyperplasia unlikely. Furthermore, the identification of discrete morular aggregates within endometrial tissue fragments supports an endometrial origin rather than simple contamination from cervical epithelium and also differs morphologically from benign squamous metaplasia, which would show gland-lining epithelium. 

Immunohistochemical analysis was not performed in this case, which represents a limitation, as it could have provided additional diagnostic support and molecular characterization of the lesion. Staining for certain markers such as p63 and cytokeratin (CK) 5/6 can help to distinguish squamous differentiation [8]. At a molecular level, morule differentiation has been associated with activation of the Wnt/β-catenin signaling pathway, particularly in lesions harboring CTNNB1 mutations [6,7]. Nuclear accumulation of β-catenin has been observed in morular structures and may represent an early molecular event in the development of endometrioid adenocarcinoma [7]. While the presence of morules alone does not establish a premalignant diagnosis, their identification may reflect underlying molecular alterations within the endometrium [2]. IHC evaluation, particularly nuclear β-catenin staining, may therefore serve as a useful adjunct in distinguishing morular differentiation from other forms of squamous change.

The patient described in this report demonstrated several established risk factors for estrogen-mediated endometrial proliferation, including morbid obesity, early menarche, and nulliparity [9,10]. Obesity contributes to increased peripheral aromatization of adrenal androgens within adipose tissue, resulting in elevated circulating estrone levels and a state of relative unopposed estrogen exposure [11]. Estrogen alterations promote endometrial proliferation and increase the risk of endometrial hyperplasia and carcinoma. Additionally, early menarche and nulliparity further increase cumulative lifetime estrogen exposure and risk of proliferative pathology.

Despite these risk factors, the endometrial biopsy demonstrated isolated SMM without evidence of proliferative pathology. However, an important limitation of endometrial biopsy is its sampling nature, as only a small portion of the endometrium is collected. Focal lesions may therefore be missed, supporting the role of clinical follow-up and repeat sampling in selected cases [12,13]. 

Management of isolated squamous morular metaplasia has not been standardized due to its rarity. In patients with risk factors for estrogen-driven proliferation, progestin therapy may represent a reasonable preventive strategy [14]. Levonorgestrel-releasing intrauterine devices exert a strong local progestogenic effect on the endometrium, leading to stromal decidualization, glandular atrophy, and suppression of endometrial proliferation. These effects contribute to reduced menstrual bleeding and decreased risk of hyperplastic progression [15,16]. 

This case highlights the importance of recognizing isolated morular metaplasia in patients with risk factors for estrogen-driven pathology, as it may serve as an early marker warranting surveillance. In addition, progestin therapy with a levonorgestrel-releasing intrauterine device may serve as a management strategy to maintain endometrial stability while minimizing the need for repeated or unnecessary endometrial biopsies in high-risk patients. However, as this report describes a single case with limited follow-up, conclusions regarding long-term clinical behavior should be interpreted with caution, and broader associations are based on previously published literature rather than the present case alone.

## Conclusions

Isolated squamous morular metaplasia is an uncommon histopathologic finding in endometrial sampling that is typically associated with endometrioid proliferative lesions but may also occur in the absence of overt pathology. In such cases, its clinical significance remains uncertain and requires careful clinical judgment to guide evaluation and follow-up. This case highlights that isolated morular metaplasia can be identified in patients with significant risk factors for estrogen-driven proliferation despite the absence of hyperplasia or malignancy. This raises concern for an endometrium that may be predisposed to future proliferative change. Given the known limitations of endometrial sampling, this finding may warrant closer surveillance rather than immediate reassurance.

In this context, progestin therapy with a levonorgestrel-releasing intrauterine device may offer a practical management strategy by promoting endometrial stability while potentially reducing the need for repeated invasive biopsies. This approach may be particularly valuable in high-risk patients where balancing adequate surveillance with avoidance of over-intervention is essential. Further studies are needed to better define the prognostic significance of isolated morular metaplasia and to clarify the role of molecular and immunohistochemical markers in guiding risk stratification and management.
